# The role of non-coding RNAs in the pathogenesis of migraine: from molecular insights to potential biomarkers

**DOI:** 10.3389/fneur.2026.1838515

**Published:** 2026-08-11

**Authors:** Li Zhang, Dijun Wang, Weiqi Wang, Juhua Chen, Hongyu Qian, Yonglan Ruan

**Affiliations:** 1 Department of Neurology, Changzhou Hospital Affiliated to Nanjing University of Chinese Medicine, Changzhou, China; 2 Changzhou Key Laboratory of Human Use Experience Research & Transformation of Menghe Medical School, Changzhou Hospital Affiliated to Nanjing University of Chinese Medicine, Changzhou, China

**Keywords:** biomarker, circular RNA, long non-coding RNA, microRNA, migraine, neuroinflammation, non-coding RNA, trigeminovascular system

## Abstract

Non-coding RNAs (ncRNAs) have emerged as critical post-transcriptional regulators in migraine pathophysiology, yet their clinical translational potential remains incompletely defined. This narrative review synthesizes current evidence on microRNAs (miRNAs), long non-coding RNAs (lncRNAs), and circular RNAs (circRNAs) in migraine, organizing findings around a “mechanism–biomarker–intervention” framework. Key findings include: (1) multiple miRNAs (e.g., miR-155, miR-34a-5p, miR-342-3p) are differentially expressed in peripheral blood mononuclear cells (PBMCs) or serum of migraine patients, with expression patterns correlating with attack phase, chronicity, and treatment response to CGRP-targeted therapies; (2) the lncRNA NEAT1 regulates photophobic behavior via the miR-196a-5p/Trpm3 axis in preclinical models, though evidence derives exclusively from male mice—a significant limitation given the 3:1 female-to-male prevalence ratio of migraine; (3) recent studies have begun to provide direct evidence for circRNA involvement in migraine, including differential circRNA expression profiles in patient PBMCs and functional characterization of hsa_circ_0006168 in promoting central sensitization; and (4) lncRNAs PVT1 and MEG3 show differential expression between migraine with and without aura, suggesting subtype-specific biomarker potential. However, the field faces substantial challenges: most studies employ small, cross-sectional designs; biological matrices are inconsistently reported; evidence is predominantly preclinical; and no ncRNA-based diagnostic or therapeutic approach has entered clinical trials. We critically appraise these limitations and outline priorities for future research, including multicenter cohort validation, standardized detection protocols, and integration of multi-omics data. While ncRNAs represent promising candidates for migraine biomarkers and therapeutic targets, their clinical translation requires rigorous validation beyond current proof-of-concept studies.

## 1 Introduction

Migraine is a highly disabling primary headache disorder with a high global prevalence that severely impacts patients’ quality of life and social functioning. Although various acute and preventive treatment options are available, a significant proportion of patients respond poorly to or cannot tolerate existing medications. Clinical management remains particularly challenging in populations with chronic migraine and medication-overuse headache (CM-MO) (1). Furthermore, migraine subtypes are complex, including both aura and non-aura types, and their pathophysiological mechanisms remain incompletely understood, leading to a lack of effective tools for precise diagnosis and personalized treatment. In current clinical practice, there is a lack of reliable objective biomarkers for early identification, subtype differentiation, prediction of chronicity risk, and assessment of treatment response. For example, studies have found that the expression levels of miR-155 in peripheral blood mononuclear cells (PBMCs) of patients with chronic migraine with medication overuse are significantly higher than those in patients with episodic migraine and healthy controls, and are positively correlated with the pro-inflammatory factors IL-1β and TNF-*α*. This suggests a key role for inflammatory mechanisms in disease progression and highlights the urgent need to develop novel diagnostic and monitoring tools based on molecular characteristics (1). Therefore, in-depth analysis of the molecular basis of migraine and the identification of translatable biomarkers have become core issues requiring urgent breakthroughs in this field.

In recent years, research on non-coding RNAs (ncRNAs) has undergone a paradigm shift from being regarded as “genomic dark matter” to being recognized as key regulatory elements. As RNA molecules that do not encode proteins—including microRNAs (miRNAs), long non-coding RNAs (lncRNAs), and circular RNAs (circRNAs)—they play central roles in epigenetic regulation, post-transcriptional modifications, and signaling pathway interactions. In neurological diseases, ncRNAs have been shown to participate in processes such as neuroinflammation, synaptic plasticity, ion channel regulation, and vascular function modulation (2–4). Multiple studies have revealed differentially expressed ncRNAs in migraine patients, including miRNAs in PBMCs and serum (e.g., miR-342-3p, miR-532-3p, miR-758-5p (5); miR-145-5p, miR-26a-5p, miR-19a-3p (6)) and the lncRNA NEAT1 in preclinical models (3). These findings expand our understanding of migraine pathogenesis and highlight the potential of ncRNAs as non-invasive, dynamic, and quantifiable biomarkers. Notably, recent studies have also begun to provide direct evidence for circRNA involvement in migraine, including differential circRNA expression profiles in patient blood samples (7) and functional characterization of specific circRNAs in promoting central sensitization (8), advancing circRNA research from speculation to preliminary experimental validation.

This article is a narrative review. We conducted a literature search in PubMed, Web of Science, and Google Scholar using the terms “migraine AND (non-coding RNA OR microRNA OR miRNA OR long non-coding RNA OR lncRNA OR circular RNA OR circRNA)” and related combinations, covering publications up to February 2026. Both clinical and preclinical studies were included; review articles and opinion pieces were used for contextual framing. No formal systematic review methodology was applied, and the selection of studies reflects the authors’ judgment of relevance and scientific quality.

Given the rapid progress and translational prospects of ncRNA research in migraine, this article aims to systematically organize current evidence and construct a knowledge map spanning from molecular mechanisms to clinical applications. This review first integrates the regulatory networks of microRNAs, lncRNAs, circRNAs, and other novel ncRNAs in migraine, focusing on their roles in core pathological processes such as activation of the trigeminovascular system, cortical spreading depression (CSD), neuroinflammation, and pain transmission (2, 3, 9, 10). Second, it conducts an in-depth analysis of the associations between ncRNA expression profiles and clinical phenotypes (such as attack frequency, aura status, chronicity, and drug responsiveness), emphasizing their value as tools for stratified diagnosis and treatment (1, 11–13). Furthermore, we will explore optimization strategies for ncRNA detection technologies in peripheral blood, cerebrospinal fluid, and exosomes, and propose a development pathway for multi-omics integrated biomarker panels (2, 6, 12). Finally, this review will look ahead to ncRNA-based therapeutic intervention strategies, including cutting-edge approaches such as RNA interference, epigenetic editing, and targeted delivery systems (10, 12), while critically appraising their current limitations, including off-target effects, safety concerns, and the absence of clinical trials. The innovation of this review lies in breaking away from the traditional research paradigm that disconnects mechanisms from clinical applications. By adopting a “mechanism–biomarker–intervention” triad as the central logical framework, it aims to facilitate the translation of ncRNA research findings into precision medicine practice, thereby providing new insights for addressing unmet clinical needs in the field of migraine.

## 2 Research progress on core non-coding RNAs in the pathogenesis of migraine

### 2.1 Regulatory networks of microRNAs in migraine

In recent years, the role of microRNAs (miRNAs) as key post-transcriptional regulatory factors in the pathogenesis of migraine has gradually been elucidated. Multiple studies, validated through high-throughput sequencing and qPCR, have identified significantly differentially expressed miRNAs in peripheral blood mononuclear cells (PBMCs) from migraine patients, with these changes exhibiting phase-specificity. For example, hsa-miR-3202 and hsa-miR-5189-3p exhibit significant differential expression during both the attack phase and the interictal phase; their predicted target genes are enriched in immune-inflammatory responses, neuroinflammation, and oxidative stress pathways, suggesting that miRNAs participate in regulating core pathological processes of migraine (2). Furthermore, the expression level of miR-155 in PBMCs from patients with chronic migraine with medication overuse (CM-MO) (2.68 ± 2.47 RQ) was significantly higher than that in the episodic migraine group (1.46 ± 0.85 RQ) and the healthy control group (0.44 ± 0.18 RQ), and it was positively correlated with the pro-inflammatory factors IL-1β and TNF-*α*, highlighting its key role in the regulation of inflammation in chronic migraine (1).

In animal models, upregulation of miR-155-5p in the trigeminal nucleus caudatus (TNC) suppresses SIRT1 expression, thereby activating microglia and exacerbating central sensitization; conversely, inhibiting miR-155-5p or activating SIRT1 effectively alleviates neuroinflammatory responses, indicating that the miRNA-SIRT1 axis is a critical regulatory pathway in chronic migraine (14). Clinical cohort studies have also found that miR-342-3p, miR-532-3p, and miR-758-5p are differentially expressed in premenopausal female migraine patients, with their target genes primarily involved in migraine-related pathways such as ion channel function, neurotransmitter and hormonal homeostasis, and circadian rhythm regulation (5). Notably, the expression dynamics of certain miRNAs are closely associated with treatment response: for example, following treatment with the anti-CGRP monoclonal antibody erenumab, the expression levels of hsa-miR-143-3p, hsa-miR-34a-5p, and hsa-miR-382-5p in circulation changed significantly in response to treatment and disease type, suggesting that they may serve as predictive biomarkers of drug responsiveness (12). Furthermore, members of the let-7 family (such as let-7a-5p, let-7b-5p, and let-7f-5p) are upregulated in patients during the acute phase, while miR-130a-3p is associated with blood–brain barrier disruption (4). It should be noted that this finding was derived from a study primarily investigating reversible cerebral vasoconstriction syndrome (RCVS), in which the authors performed cross-validation in a cohort of migraine patients; therefore, the let-7 upregulation data represent cross-disease validation rather than evidence from a primary migraine study. Serum miRNA profiles also show abnormalities, such as upregulation of miR-145-5p and miR-26a-5p, while miR-19a-3p is downregulated during attacks (6). Large-scale sequencing studies have further identified miR-183, miR-25, and miR-320 as being commonly altered in migraine patients, whereas miR-1307-5p, miR-6810-5p, let-7e, and miR-140-3p were specifically altered only during the attack phase. Functional analyses pointed to the involvement of prolactin and estrogen signaling pathways, and a composite diagnostic model with a positive predictive value exceeding 90% was constructed (13). However, this PPV was derived from a case–control study enriched with migraineurs versus healthy controls; PPV is prevalence-dependent, and this value may not reflect real-world clinical performance in the general population.

Beyond these high-throughput profiling studies, a growing body of human clinical research has systematically characterized individual miRNAs across diverse biological matrices, revealing a temporal trajectory of discovery from pioneering profiling efforts to mechanistic and therapeutic investigations. The earliest human miRNA profiling study in migraine was conducted by Tafuri et al. (2015), who performed plasma exosomal miRNA profiling in 15 female migraine without aura (MWoA) patients versus 13 healthy controls. This pilot study identified four differentially expressed miRNAs: miR-27b was significantly upregulated, while miR-181a, let-7b, and miR-22 were downregulated in migraineurs. Notably, a logistic regression model based on this four-miRNA panel achieved an AUC of 0.956 (*p* < 0.001) for identifying migraine, with sensitivity and specificity comparable to clinical diagnostic criteria. This study also provided the first evidence linking migraine-associated miRNAs to cardiovascular risk, as several of the identified miRNAs (particularly let-7b and miR-181a) are known regulators of vascular inflammation and atherosclerosis (15). Building upon this pioneering work, Andersen et al. (16) conducted the first serum miRNA profiling study comparing migraine attack and pain-free periods. In a cohort of migraineurs examined during both ictal and interictal phases versus healthy controls, they identified miR-34a-5p, miR-29c-5p, and miR-382-5p as significantly upregulated during attacks. Critically, miR-382-5p remained elevated during the interictal period, suggesting that certain miRNAs may serve as persistent biomarkers of migraine susceptibility rather than merely reflecting acute attack status. *In silico* analysis predicted that miR-34a-5p targets GABBR2, SLC6A1, and GABRA3, while miR-382-5p targets IL10RA and GABRA5, implicating both GABAergic signaling and anti-inflammatory pathways in migraine pathophysiology (16). These two early studies established the feasibility of circulating miRNA profiling in migraine and laid the groundwork for subsequent mechanistic investigations.

A particularly important line of research has emerged around the CGRP–miRNA regulatory axis, directly connecting miRNAs to the most validated therapeutic target in migraine. Zhai and Zhu (17) demonstrated that miR-30a expression was significantly reduced in the peripheral blood of migraine patients, accompanied by elevated promoter methylation levels of the miR-30a gene. Mechanistically, miR-30a was shown to directly target and degrade CALCA mRNA—the gene encoding *α*-CGRP—thereby reducing CGRP production. Clinically, reduced miR-30a levels were associated with bilateral attacks, prolonged pain duration, and higher pain intensity indices, suggesting that the miR-30a–CALCA (CGRP) axis may serve as both a mechanistic link and a potential therapeutic target (17). This finding is particularly significant as it provides the first direct evidence that a specific miRNA can regulate CGRP at the post-transcriptional level, complementing the existing understanding of CGRP pathway regulation through epigenetic mechanisms (9). Extending this CGRP–miRNA connection, Greco et al. (2020) evaluated plasma CGRP levels alongside miR-382-5p and miR-34a-5p expression in PBMCs from 27 episodic migraine (EM) and 28 chronic migraine with medication overuse (CM-MO) patients. They found that CGRP, miR-382-5p, and miR-34a-5p were all significantly higher in CM-MO compared to EM patients (*p* = 0.003 for all comparisons), and that CGRP levels positively correlated with both miR-382-5p (Spearman’s rho = 0.491, *p* = 0.001) and miR-34a-5p (Spearman’s rho = 0.303, *p* = 0.025). Following a two-month in-hospital detoxification protocol, both CGRP and miRNA levels significantly decreased (p = 0.001), demonstrating that these biomarkers are dynamically modulated by treatment. This study was the first to propose a combined CGRP + miRNA biomarker panel for migraine, bridging the gap between neuropeptide and epigenetic biomarker research (18). The convergence of these findings with the existing observation that erenumab treatment modulates miR-143-3p, miR-34a-5p, and miR-382-5p expression (12) underscores the intimate coupling between the CGRP pathway and miRNA regulation in migraine.

In addition to the CGRP axis, endothelial dysfunction—a well-documented component of migraine vascular pathophysiology—has been linked to specific miRNA signatures. Cheng et al. (19) conducted the first investigation of endothelial-specific miRNAs in migraine, quantifying circulating levels of miR-155, miR-126, miR-21, and let-7 g in 30 migraine patients versus 30 matched healthy controls. They found that miR-155 (6.17-fold, *p* = 0.018), miR-126 (6.17-fold, *p* = 0.013), and let-7 g (7.37-fold, *p* = 0.005) were significantly elevated in migraineurs, while miR-21 showed no significant difference. Notably, miR-155 and miR-126 levels were positively correlated with syncope frequency (*r* = 0.375, *p* = 0.041 for both), and patients with migraine with aura exhibited insignificantly higher miRNA levels than those without aura. This study was the first to link endothelial-specific miRNAs to migraine vascular mechanisms, suggesting that miR-155, miR-126, and let-7 g may serve as biomarkers reflecting the endothelial dysfunction component of migraine pathophysiology (19). The finding that miR-155 is elevated in plasma is consistent with its previously reported upregulation in PBMCs of CM-MO patients (1), suggesting that this miRNA may operate across multiple biological compartments and cell types.

The question of cell-type specificity has been directly addressed by recent advances in monocyte-focused research. Greco et al. (2025) measured miR-382-5p and miR-34a expression specifically in monocytes from 47 EM patients, 32 CM-MO patients, and 30 healthy controls. They found that both miRNAs were significantly elevated in monocytes of EM and CM-MO patients compared to healthy controls (*p* = 0.005), and that CM-MO patients showed higher levels than EM patients (*p* = 0.005), with expression levels correlating with disease severity. A post-hoc exploratory comparison with PBMC expression data from their previous study (18) revealed that while both cell populations showed elevated miRNA expression in migraine, the magnitude of differential expression was more pronounced in the purified monocyte fraction, highlighting the importance of cell-type-specific analysis. This study represents the first direct validation of miR-382-5p and miR-34a in a specific immune cell subpopulation, building upon the earlier finding that miR-155 is differentially expressed in monocytes of migraine patients (1) and revealing that different miRNAs may have distinct cell-type-specific regulatory roles in migraine (20).

The therapeutic implications of miRNA dysregulation have been further explored in the context of anti-CGRP treatment. De Icco et al. (21) conducted an open-label study of 40 chronic migraine patients treated with erenumab (70 mg subcutaneously every 28 days for three administrations), measuring miR-382-5p and miR-34a-5p levels in PBMCs before and after 12 weeks of treatment. They found that both miR-382-5p (*p* = 0.015) and miR-34a-5p (*p* = 0.001) were significantly reduced following erenumab treatment in the overall population. Neurophysiologically, responders (≥30% reduction in monthly migraine days) showed significant increases in both reflex threshold (RTh) and temporal summation threshold (TST) (*p* = 0.001 for both), while non-responders did not. Importantly, miRNA changes did not differ significantly between responders and non-responders, suggesting that miRNA modulation may reflect a general pharmacological effect of CGRP pathway blockade rather than being solely tied to clinical response. This finding complements the work of Ornello et al. (12), who reported that miR-143-3p, miR-34a-5p, and miR-382-5p levels changed dynamically with erenumab treatment in a women-only cohort, and together these studies suggest that different migraine phenotypes may exhibit distinct treatment susceptibility profiles at both the neurophysiological and biomolecular levels (21).

It is worth noting that hsa-miR-7855-5p, identified as differentially expressed in PBMCs from migraine patients (2), has been confirmed to exist in miRBase v22.1 (accession MI0025525, MIMAT0030430), verifying that the nomenclature is correct (Table 1).

**Table 1:** Summary of key miRNAs implicated in migraine pathogenesis.

miRNA	Biological matrix	Key findings	Molecular pathway	Evidence type	Ref.
miR-155	PBMCs (human)	Upregulated in CM-MO vs. EM vs. HC; correlated with IL-1β, TNF-α	Inflammation/ SIRT1	Clinical + Preclinical	(1, 14)
miR-342-3p, miR-532-3p, miR-758-5p	PBMCs (human)	Differentially expressed in Premenopausal female migraine patients	Ion channels, neurotransmitter homeostasis, circadian rhythm	Clinical	(5)
miR-143-3p, miR-34a-5p, miR-382-5p	Serum (human)	Changed after erenumab treatment; baseline differences CM vs. EM	CGRP pathway	Clinical	(12)
miR-145-5p, miR-26a-5p	Serum (human)	Upregulated during attacks	Unknown	Clinical	(6)
miR-19a-3p	Serum (human)	Downregulated during attacks	Unknown	Clinical	(6)
miR-3202, miR-5189-3p, miR-7855-5p	PBMCs (human)	Differentially expressed attack vs. interictal	Immune inflammation, neuroinflammation, oxidative stress	Clinical	(2)
let-7a-5p, let-7b-5p, let-7f-5p	Plasma (human)	Upregulated in acute phase (RCVS study with migraine cross-validation)	Unknown	Clinical (cross-disease)	(4)
miR-183, miR-25, miR-320	Blood (human)	Commonly altered; composite model PPV > 90%	Prolactin, estrogen signaling	Clinical	(13)
miR-653-3p	Rat model	Targets IGF1; alleviates NO₂-induced migraine	AKT/TRPV1	Preclinical	(24)
miR-34a-5p	Rat model (TNC)	Upregulated via METTL3/m6A; Inhibits Wnt1/*β*-catenin	Wnt/β-catenin	Preclinical	(10)
miR-27b, miR-22, miR-181a, let-7b	Plasma exosomes (human)	↑miR-27b; ↓miR-181a, let-7b, miR-22 in MWoA; AUC = 0.956	Cardiovascular risk	Clinical	(15)
miR-34a-5p, miR-29c-5p, miR-382-5p	Serum (human)	↑during attacks; miR-382-5p ↑interictally	GABAergic signaling, IL-10RA	Clinical	(16)
miR-30a	Peripheral blood (human)	↓in migraine;promoter hyper-methylation; targets CALCA	CALCA/ CGRP	Clinical	(17)
miR-382-5p, miR-34a-5p	PBMCs (human)	↑in CM-MO vs. EM; Correlated with CGRP; ↓after detox	CGRP pathway	Clinical	(18)
miR-155, miR-126, let-7 g	Plasma (human)	↑in migraine (endothelial- specific); correlated with syncope	Endothelial dysfunction	Clinical	(19)
miR-382-5p, miR-34a	Monocytes (human)	↑in EM and CM-MO vs. HC; ↑CM-MO vs. EM; Correlated with severity	CGRP pathway	Clinical	(20)
miR-382-5p, miR-34a-5p	PBMCs (human)	↓after erenumab treatment; no difference responders vs. non-responders	CGRP pathway	Clinical	(21)

### 2.2 Epigenetic regulatory roles of long non-coding RNAs

Long non-coding RNAs (lncRNAs) regulate gene expression through epigenetic mechanisms and exhibit potential roles in the pathophysiology of migraine. An animal study found that in a male mouse model of migraine-associated photophobic behavior, the expression of the lncRNA NEAT1 was significantly upregulated within the trigeminal ganglion; NEAT1 competitively binds to miR-196a-5p, thereby lifting its inhibitory effect on Trpm3, which in turn upregulates Trpm3 levels and promotes photophobic-like behavior; conversely, downregulation of NEAT1 effectively alleviated this behavior, revealing the regulatory mechanism of the NEAT1/miR-196a-5p/Trpm3 axis in migraine sensory sensitization (3). It is important to note that this evidence is derived from a male-only mouse model. Given that migraine exhibits an approximately 3:1 female-to-male prevalence ratio in humans, the generalizability of these findings to female patients remains uncertain and requires direct validation in female animal models. Mechanistically, NEAT1 functions as a nuclear paraspeckle-associated lncRNA that serves as a competing endogenous RNA (ceRNA): by sequestering miR-196a-5p through complementary base-pairing, NEAT1 prevents miR-196a-5p from binding to and degrading Trpm3 mRNA, thereby relieving post-transcriptional repression of Trpm3. The resulting increase in TRPM3 channel expression enhances excitability of trigeminal sensory neurons, promoting photophobic behavior. This ceRNA mechanism represents a specific example of how lncRNAs can fine-tune ion channel expression in the trigeminal pain pathway.

Although direct lncRNA research on migraine remains limited, studies on other neurological disorders provide important clues. For example, in a mouse model of traumatic brain injury, astrocyte-derived exosomes carrying the lncRNA 4933431K23Rik act as “sponges” to bind miR-10a-5p, thereby upregulating E2F7 and TFAP2C expression. This ultimately enhances Smad7 expression to inhibit NF-κB signaling pathway activation and alleviate neuroinflammation (22). This finding was derived from a traumatic brain injury (TBI) mouse model, not a migraine model; its relevance to migraine is speculative and requires direct validation. This mechanism suggests that lncRNAs may participate in the central sensitization process of migraine by regulating neuroinflammatory pathways. Furthermore, existing studies have shown that alterations in DNA methylation and histone deacetylase activity are closely associated with migraine chronicity, TRPV1/TRPA1 signaling pathways, and hormone receptor signaling pathways; as lncRNAs serve as important mediators of epigenetic regulation, they may play a bridging role in these processes (11).

Recent clinical evidence has further expanded the lncRNA landscape in migraine. Taheri et al. (23) measured the expression of five circulating lncRNAs—PVT1, DSCAM-AS1, MEG3, LINC-ROR, and SPRY4-IT1—in the blood of migraine patients. PVT1 and MEG3 were significantly upregulated in migraineurs compared to healthy controls. Notably, both PVT1 (*p* < 0.0001) and MEG3 (*p* = 0.01) showed differential expression between migraine with aura (MA) and migraine without aura (MO), suggesting subtype-specific regulatory roles. PVT1 demonstrated particularly strong discriminative ability for MA versus controls (AUC = 0.98), highlighting its potential as a diagnostic biomarker for aura-associated migraine (23). These findings represent some of the first direct clinical evidence linking specific lncRNAs to migraine subtype differentiation (Table 2).

**Table 2:** Summary of key lncRNAs implicated in migraine or related neuroinflammatory processes.

lncRNA	Biological matrix	Key findings	Molecular pathway	Evidence type	Ref.
NEAT1	Trigeminal ganglion (male mouse)	Upregulated; regulates photophobia via miR-196a-5p/Trpm3	ceRNA/miR-196a-5p/Trpm3	Preclinical (male-only model)	(3)
4933431K23Rik	Astrocyte exosomes (TBI mouse)	Suppresses NF-κB via miR-10a-5p/E2F7/TFAP2C	NF-κB/SMAD7	Preclinical (TBI model, not migraine)	(22)
PVT1	Whole blood (human)	Upregulated in migraine; differs MA vs. MO (*p* < 0.0001); AUC = 0.98 for MA vs. control	Unknown	Clinical	(23)
MEG3	Whole blood (human)	Upregulated in migraine; differs MA vs. MO (*p* = 0.01)	Unknown	Clinical	(23)
DSCAM-AS1, LINC-ROR, SPRY4-IT1	Whole blood (human)	Measured in migraineurs; no significant differences reported	Unknown	Clinical	(23)
Nostrill	Microglia (mouse, LPS-stimulated)	Promotes NF-κB/iNOS; enhances H3K4me3	NF-κB/iNOS	Preclinical (not migraine-specific)	(42)

### 2.3 The ceRNA mechanism of circular RNAs and their interaction with signaling pathways

Circular RNAs (circRNAs), acting as competitive endogenous RNAs (ceRNAs), can regulate the expression of downstream target genes by “sponge-like” binding of miRNAs. While research on their role in migraine was previously limited to indirect evidence from other diseases, recent studies have begun to provide direct experimental data supporting circRNA involvement in migraine pathophysiology.

Lin et al. (2020) conducted the first differential expression analysis of circRNAs in migraine patients, comparing 4 migraine patients with 3 healthy controls. This study identified 2,039 differentially expressed circRNAs, with pathway enrichment analysis revealing significant involvement of the PI3K-Akt signaling pathway—a pathway previously implicated in migraine through the miR-653-3p/IGF1/AKT axis (24). Although the sample size was small, this study provided the first catalog of circRNA dysregulation in migraine patients and established a foundation for subsequent functional investigations (7).

More recently, Chen et al. (2026) provided the first direct functional evidence for a specific circRNA in migraine. They demonstrated that hsa_circ_0006168 is upregulated in the trigeminal nucleus caudalis (TNC) in a mouse model of migraine and drives microglial activation through the miR-99b-5p/KDM6B axis, ultimately promoting central sensitization. This study represents a critical advance, as it moves circRNA research in migraine from observational expression profiling to mechanistic functional validation, establishing circRNAs as active participants rather than passive biomarkers in migraine pathophysiology (8).

In parallel, relevant mechanisms have been well-validated in other diseases. For example, in oral cancer, circRNAs regulate processes such as inflammatory responses and tumor metastasis by forming ceRNA networks (19); in head and neck cancers, circRNAs are being explored as biomarkers for liquid biopsy (25). In the context of migraine research, existing evidence suggests that non-coding RNAs can influence the pathological process of migraine by regulating the epigenetic modifications of the TRPA1 and CGRP genes in the RONS-TRPA1-CGRP axis (9), and it is plausible that circRNAs participate in the regulation of this axis via the ceRNA mechanism. Furthermore, NEAT1, as a lncRNA, has been shown to regulate the miR-196a-5p/Trpm3 pathway via the ceRNA mechanism (3), suggesting that circRNAs may also employ similar strategies to interfere with migraine-related ion channels or neuropeptide signaling pathways. Future studies should systematically identify differentially expressed circRNAs in larger cohorts of migraine patients and further elucidate their interactions with miRNA-mRNA regulatory networks (Table 3).

**Table 3:** Summary of circRNAs implicated in migraine.

circRNA	Biological matrix	Key findings	Molecular pathway	Evidence type	Ref.
2,039 DE circRNAs (panel)	PBMCs (human)	4 migraine vs. 3 controls; enriched in PI3K-Akt pathway	PI3K-Akt	Clinical (small sample)	(7)
hsa_circ_0006168	TNC (mouse model)	Drives microglial activation; promotes central sensitization	miR-99b-5p/KDM6B	Preclinical	(8)

### 2.4 Potential contributions of other non-coding RNAs (tRNA fragments, snoRNAs, etc.)

Apart from miRNAs, lncRNAs, and circRNAs, the roles of other types of non-coding RNAs, such as tRNA-derived fragments (tRFs) and small nucleolar RNAs (snoRNAs), in migraine have not yet been directly studied; however, their potential contributions should not be overlooked. Existing literature indicates that non-coding RNAs as a whole may participate in regulating cellular redox balance by influencing epigenetic mechanisms, and oxidative stress is a key pathological component of migraine (26). Furthermore, m^6^A RNA methylation, a key mechanism in epigenetic transcriptome regulation, can be modulated by demethylases such as ALKBH5, thereby influencing the stability and function of various non-coding RNAs, including miRNAs, lncRNAs, and circRNAs (27). Although these studies have primarily focused on the field of oncology (26, 27), given that abnormalities in epigenetic regulation have been observed in migraine (11), tRFs and snoRNAs may participate in the disease process through similar mechanisms. For example, snoRNAs can guide rRNA modification, affecting protein translation efficiency, while tRFs have been shown to regulate gene silencing and stress responses. Future studies should utilize multi-omics technologies to systematically screen the expression profiles of these non-canonical non-coding RNAs in migraine patients to expand our understanding of the molecular mechanisms underlying migraine.

## 3 Studies on the association between non-coding RNAs and clinical features of migraine

### 3.1 Molecular biomarkers of attack frequency and duration

The expression levels of non-coding RNAs are closely associated with the frequency and duration of migraine attacks. Studies have shown that changes in the expression of circulating miRNAs are significantly correlated with migraine attack characteristics, and their levels tend to decrease following treatment with gepant-class drugs that target the CGRP pathway, suggesting that these molecules may serve as dynamic indicators of disease activity (11). Furthermore, in a cohort study including patients with acute, interictal, and chronic migraine, miR-183, miR-25, and miR-320 were commonly abnormally expressed in migraine patients, whereas miR-1307-5p, miR-6810-5p, let-7e, and miR-140-3p showed significant differential expression only in patients during the acute phase, suggesting that specific miRNAs may be closely associated with the acute attack state (13). However, the composite diagnostic model achieving PPV > 90% in this study was derived from a case–control design enriched with migraineurs versus healthy controls; PPV is prevalence-dependent, and this value may not reflect real-world clinical performance in the general population. Notably, miR-19a-3p is downregulated during the attack phase compared to the interictal phase, further supporting its potential as a biomarker related to the attack course (6). These findings collectively suggest that dynamic changes in specific non-coding RNAs may serve as potential molecular markers for assessing the frequency and duration of migraine attacks.

### 3.2 Differential expression profiles between aura and non-aura subtypes

Although some studies have failed to observe significant differences in non-coding RNA expression between different migraine subtypes (6), a growing body of evidence suggests that certain non-coding RNAs may be involved in the pathological differentiation between aura (MA) and non-aura (MO) migraine. For example, non-coding RNAs that regulate alternative RNA processing of the CALCA gene may participate in the neuroplasticity processes of migraine subtypes by influencing CGRP generation, thereby forming the basis of subtype-specific regulatory mechanisms (9). Furthermore, non-coding genetic variants located within regulatory elements (such as VAMP2*rs1150 and SNAP25*rs2327264) may influence gene expression by interfering with microRNA binding, thereby contributing to migraine susceptibility and subtype differentiation (28).

Importantly, Taheri et al. (23) provided direct clinical evidence for lncRNA dysregulation between migraine subtypes. Among the five circulating lncRNAs examined, PVT1 showed highly significant differential expression between MA and MO (*p* < 0.0001), while MEG3 also reached statistical significance (*p* = 0.01). PVT1 demonstrated an AUC of 0.98 for distinguishing MA from healthy controls, suggesting strong diagnostic potential for aura-specific migraine. These findings indicate that lncRNAs may not only serve as general migraine biomarkers but also contribute to the molecular basis of aura-specific pathophysiology, possibly through involvement in cortical spreading depression-related cascades or neurovascular coupling mechanisms (23).

Although large-scale studies directly comparing the non-coding RNA expression profiles of MA and MO patients are currently lacking, functional pathway enrichment analyses indicate that miRNAs associated with ion channels, neurotransmitter homeostasis, and inflammatory responses (such as miR-342-3p, miR-532-3p, and miR-758-5p) are significantly dysregulated in migraine patients, and these pathways are also closely related to the pathophysiology of migraine (5). Therefore, future studies should systematically analyze the differential expression patterns of non-coding RNAs in MA and MO through larger sample sizes and more refined subtype stratification.

### 3.3 Key regulatory factors in the transition to chronicity

During the transition of migraine from episodic (EM) to chronic (CM), the expression of specific non-coding RNAs undergoes significant changes, suggesting their key regulatory role in disease progression. A cross-sectional study found that the expression level of miR-155 in PBMCs was significantly higher in patients with CM-MO compared to those with EM and healthy controls, and was positively correlated with the pro-inflammatory factors IL-1β and TNF-*α*, suggesting that it may promote chronicity by driving neuroinflammation (detailed numerical data are presented in Section 2.1) (1). Furthermore, in patients treated with erenumab, baseline levels of hsa-miR-34a-5p and hsa-miR-382-5p were significantly higher in CM patients than in EM patients, and these levels decreased significantly after treatment, suggesting that these miRNAs may be involved in the maintenance mechanisms of chronic migraine (12). Concurrently, the epigenetic regulation of the CALCA/CGRP axis and the TRPA1/RONS signaling pathway by non-coding RNAs is also believed to play a crucial role in the neuroplastic remodeling associated with migraine chronicization (9). Taken together, these findings point to miR-155, miR-34a-5p, and miR-382-5p as key regulatory factors and potential intervention targets in the transition to chronic migraine.

### 3.4 Predictive biomarkers for drug response

Non-coding RNAs show great promise in predicting treatment response in migraine. Studies have shown that after 16 weeks of treatment with erenumab, the expression of hsa-miR-143-3p in EM patients gradually decreased as treatment response improved (*p* = 0.02), while hsa-miR-34a-5p and hsa-miR-382-5p levels in CM patients significantly decreased following treatment (*p* = 0.0002, *p* = 0.0007; *p* = 0.04, p = 0.02), suggesting that these miRNAs may serve as dynamic predictive markers of the efficacy of CGRP-targeted therapies (12). Furthermore, circulating miRNA levels also showed a downward trend following treatment with gepants, further supporting their potential as biomarkers of treatment response (11). Genetic risk scoring models based on miRNA expression have effectively distinguished migraine patients from healthy controls, with a positive predictive value exceeding 90% (13), however, this PPV was derived from a case–control study enriched with migraineurs versus healthy controls; PPV is prevalence-dependent, and this value may not reflect real-world clinical performance in the general population. Overall, specific non-coding RNAs not only reflect disease status but also enable dynamic monitoring of treatment response, offering promise as important predictive tools for guiding precision medicine.

## 4 Key pathways and molecular regulatory mechanisms

### 4.1 RNA regulatory networks in the trigeminovascular system

The trigeminovascular system is the core anatomical and functional unit for migraine pain signaling; its activation triggers key pathological processes such as neurogenic inflammation, vasodilation, and central sensitization. In recent years, non-coding RNAs (ncRNAs) have been shown to exert multi-level regulatory roles within this system. Studies have found that in a rat migraine model induced by chronic intermittent nitroglycerin, the expression of N6-methyladenosine (m^6^A) methyltransferase METTL3 is upregulated. By enhancing the expression of miR-34a-5p, it inhibits the Wnt1/*β*-catenin signaling pathway, thereby promoting neuroinflammation and migraine-like behavior. This reveals a new mechanism by which epigenetic modifications and miRNAs synergistically regulate trigeminovascular system function (10).

Furthermore, in the trigeminal ganglia of male mice, elevated expression of the long non-coding RNA NEAT1 acts as a competitive endogenous RNA (ceRNA) to bind miR-196a-5p, thereby lifting its inhibition of Trpm3 and enhancing photophobic-like behavior; inhibition of NEAT1 or TRPM3 significantly alleviates this behavior, indicating the critical role of the lncRNA-miRNA-mRNA axis in the regulation of trigeminal sensory function (3). This evidence is derived from a male-only mouse model; given the approximately 3:1 female-to-male prevalence ratio of migraine in humans, these findings require validation in female models before generalization. Other studies have indicated that non-coding RNAs can influence the production of calcitonin gene-related peptide (CGRP)—a key mediator in trigeminovascular system activation and migraine attacks—by regulating the post-transcriptional processing of the CALCA gene, suggesting that ncRNAs may participate in the pathophysiological process of migraine by intervening in the RONS-TRPA1-CGRP signaling axis (9). Analysis of clinical samples further supports this view: serum levels of miR-145-5p and miR-26a-5p are significantly elevated in migraine patients, and miR-19a-3p expression is lower during attacks than during remission (6); Multiple differentially expressed miRNAs (e.g., hsa-miR-3202, hsa-miR-7855-5p) were also detected in peripheral blood mononuclear cells (PBMCs), with their target genes enriched in immune inflammation, neuroinflammation, and oxidative stress pathways (2). These findings collectively construct a trigeminovascular regulatory network centered on ncRNAs, providing a molecular basis for understanding the peripheral initiation mechanisms of migraine.

### 4.2 ncRNA regulatory mechanisms of cortical spreading depression

Cortical spreading depression (CSD) is considered the central pathophysiological event in migraine with aura (MWA), characterized by slow depolarization waves in neurons and glial cells, accompanied by local blood flow changes and neuroinflammatory activation. Although CSD primarily occurs in the cerebral cortex, it can subsequently activate the trigeminovascular system, triggering headaches (29). Currently, direct evidence regarding the regulatory role of ncRNAs in CSD remains limited, but indirect clues are increasing. Imaging studies have shown activation of astrocytes and dural macrophages in patients with MWA, suggesting a bridging role for neuroinflammation in the transition from CSD to pain (30). Notably, several studies have linked ncRNAs to pathways associated with CSD. For example, miR-653-3p regulates the AKT/TRPV1 signaling pathway by targeting IGF1 and is involved in nitric oxide (NO)-induced migraine models, with NO being a known effective inducer of CSD (24). Furthermore, ATG7—an autophagy-related gene associated with migraine in both the trigeminal ganglion and the cortex—exhibits upregulated expression that enhances autophagy flux, manifested in the S1HL brain region by elevated LC3-II and reduced p62 levels, suggesting that autophagy may play a role in the regulation of cortical excitability, a process that may be indirectly modulated by ncRNAs (31). Although current evidence does not explicitly describe the real-time regulation of specific ncRNAs in CSD wave propagation, given that CSD involves ion homeostasis imbalances, glutamate release, and energy metabolism disorders, and considering that multiple differentially expressed miRNAs (such as miR-342-3p, miR-532-3p) are enriched in ion channel and neurotransmitter homeostasis pathways (5), it is hypothesized that ncRNAs may indirectly influence CSD susceptibility by maintaining the cortical excitatory-inhibitory balance. Future studies should combine *in vivo* electrophysiology with spatial transcriptomics to elucidate the dynamic expression profiles and functions of ncRNAs during CSD.

### 4.3 Epigenetic basis of neuroinflammation and central sensitization

Neuroinflammation and central sensitization are key mechanisms underlying the chronicity of migraine and persistent pain. Clinical studies have shown elevated levels of pro-inflammatory cytokines (such as IL-1β, IL-6, and TNF-*α*) in the serum and cerebrospinal fluid of migraine patients, which may lead to a lowered pain threshold and hyperalgesia by enhancing the reactivity of central pain transmission pathways (32). Activation of the NLRP3 inflammasome promotes the release of these inflammatory factors and stimulates trigeminal neurons, contributing to trigeminovascular system activation and pain generation (33). In this context, non-coding RNAs, acting as epigenetic regulators, have been shown to mediate neuroinflammatory responses. The METTL3/m^6^A/miR-34a-5p axis exacerbates neuroinflammation by inhibiting the Wnt1/*β*-catenin pathway (10); meanwhile, the NEAT1/miR-196a-5p/Trpm3 network may also indirectly influence the inflammatory microenvironment by regulating the excitability of sensory neurons (3). It should be noted that this NEAT1 evidence is derived from a male-only mouse model; the 3:1 female-to-male prevalence ratio of migraine in humans limits the direct generalizability of these findings. Furthermore, the kynurenine pathway (KP) within the tryptophan metabolism pathway participates in inflammation regulation by acting on glutamate receptors, the aryl hydrocarbon receptor (AhR), and GPR35, and may interact with CGRP and PACAP (32); however, the expression of KP-related enzymes may be regulated by miRNAs, although current evidence does not clearly establish this link. Regarding central sensitization, mitochondrial damage in the thalamus has been observed in mouse models of chronic migraine and is associated with hyperalgesia; intervention with urotan A can partially reverse this phenotype (34), suggesting that energy metabolism disorders may underlie sensitization. Notably, the target genes of several circulating miRNAs (such as miR-342-3p and miR-758-5p) are involved in circadian rhythms and hormonal homeostasis (5), and circadian rhythm disruption has been shown to affect the threshold for central sensitization. In summary, ncRNAs play multiple roles in the epigenetic regulation of neuroinflammation and central sensitization by modulating the release of inflammatory factors, metabolic pathways, and neuronal plasticity.

### 4.4 RNA interference mechanisms in pain signal transduction pathways

Pain signals are transmitted from peripheral trigeminal nerve terminals through the trigeminal nucleus (TNC) to the thalamus and higher cortical regions; this process involves the precise regulation of various neuropeptides, ion channels, and second messenger systems. CGRP and PACAP are the most critical neurotransmitters in this process; they are highly expressed in trigeminal ganglion neurons and released at peripheral and central terminals, triggering peripheral and central sensitization (35, 36). Non-coding RNAs can regulate pain signal transmission by interfering with the expression or function of these key molecules. For example, miR-653-3p inhibits the AKT/TRPV1 pathway by targeting IGF1, thereby alleviating NO-induced migraine-like behavior (24); while the expression level of NPFFR2 directly influences CGRP levels in the trigeminal ganglion; its overexpression increases CGRP, whereas its inhibition decreases CGRP (37). Although it remains unclear whether NPFFR2 is regulated by ncRNA, this pathway provides a potential target for RNA interference. Furthermore, the GABAergic inhibitory system also plays a regulatory role in the trigeminovascular system: downregulation of GAD65 expression in the trigeminal ganglion in a chronic migraine model leads to reduced GABA synthesis, promoting system hyperactivity, whereas a positive allosteric modulator targeting α6GABAR effectively alleviates hyperalgesia (38), suggesting that GABA-related genes may serve as targets for miRNA regulation. Clinically, treatment with gepants (CGRP receptor antagonists) is associated with decreased levels of certain miRNAs in circulation, and the miRNA expression profile can predict the efficacy of anti-CGRP monoclonal antibodies (such as erenumab) (11), indirectly reflecting the association between ncRNAs and the drug response in pain pathways. Taken together, ncRNAs form a multi-tiered RNA interference network by targeting the CGRP/PACAP system, TRP channels, GABA synthetase, and downstream signaling molecules (such as AKT and Wnt/*β*-catenin), thereby finely regulating the generation and transmission of migraine pain signals.

## 5 Translational research on non-coding RNAs as biomarkers

### 5.1 Technical optimization of peripheral blood ncRNA detection

As a non-invasive sample source, peripheral blood holds significant value in the study of migraine-related non-coding RNA (ncRNA) biomarkers. Multiple studies have utilized peripheral blood mononuclear cells (PBMCs) or serum to conduct miRNA expression profiling. For example, using small RNA sequencing technology, researchers identified miRNAs differentially expressed between attack and interictal phases in PBMCs from migraine patients. Thirty-one miRNAs were abnormally expressed during the interictal phase, while five showed significant changes during the attack phase; these miRNAs were enriched in immune inflammation, neuroinflammation, and oxidative stress pathways (2). Furthermore, microarray analysis in a cohort of premenopausal women revealed that the triplet of miR-342-3p, miR-532-3p, and miR-758-5p was differentially expressed in PBMCs, with their target genes involved in migraine-related pathways such as ion channel regulation and neurotransmitter homeostasis (5). Technologically, SYBR qPCR combined with TaqMan qPCR has been used to validate changes in the expression of miR-145-5p, miR-26a-5p, and miR-19a-3p in serum (6); while next-generation sequencing combined with quantitative PCR has successfully identified miRNAs such as miR-183, miR-25, and miR-320 that are commonly abnormally expressed in migraine patients (13). It is worth noting that serum miRNA levels decreased following treatment with gepants, suggesting that detection methods must account for the impact of treatment status on ncRNA expression (11). The aforementioned studies indicate that a strategy combining high-throughput sequencing with targeted qPCR validation can effectively enhance the sensitivity and specificity of ncRNA detection in peripheral blood.

### 5.2 Screening strategies for specific biomarkers in cerebrospinal fluid

Although cerebrospinal fluid (CSF) collection is an invasive procedure, it provides a closer reflection of the central nervous system microenvironment and may yield more specific ncRNA biomarkers. While no large-scale studies specifically targeting ncRNAs in the CSF of migraine patients have been reported to date, research on the underlying mechanisms provides a theoretical basis for the screening of CSF biomarkers. For example, in a migraine-associated photosensitive male mouse model, the expression of the long non-coding RNA NEAT1 was upregulated within the trigeminal ganglion and regulated photosensitive behavior via the NEAT1/miR-196a-5p/Trpm3 axis (3). This evidence is derived from a male-only mouse model; the 3:1 female-to-male prevalence ratio of migraine in humans limits the direct generalizability of these findings. Furthermore, a study integrating human trigeminal ganglion single-cell RNA sequencing with cortical quantitative trait locus (QTL) data identified migraine-associated genes such as ATG7, whose regulation may involve ncRNA-mediated autophagy mechanisms; if such molecules can be stably detected in CSF, they have the potential to serve as specific biomarkers (31). Future CSF ncRNA screening should combine high-depth sequencing, rigorous control designs, and longitudinal sampling to distinguish migraine subtypes and disease stages, while excluding interference from other neurological disorders.

### 5.3 Methods for enrichment and detection of exosomal ncRNA

As carriers of intercellular communication, exosomes can transport stable ncRNAs with tissue-specific characteristics, making them ideal targets for liquid biopsy. In other neurological or chronic diseases, circulating exosomal ncRNAs have been shown to possess prognostic value; for example, levels of differentiation-inhibiting non-coding RNAs in exosomes from patients with chronic hepatitis C-associated hepatocellular carcinoma are significantly associated with tumor recurrence and mortality (39). Although systematic studies on exosomal ncRNA in the field of migraine are still lacking, existing evidence indicates that serum miRNAs (such as miR-143-3p, miR-34a-5p, and miR-382-5p) undergo dynamic changes following CGRP pathway-targeted therapy, suggesting that these changes in circulating miRNAs may reflect the dynamics of various carriers, including exosomes (12). The detection of exosomal ncRNAs relies on standardized isolation methods (such as ultracentrifugation, polymer precipitation, or immunoaffinity capture) combined with downstream small RNA sequencing or qPCR analysis. Given that exosomes can cross the blood–brain barrier and carry centrally derived ncRNAs, they offer unique advantages in the development of migraine biomarkers, particularly for monitoring molecular events related to the trigeminovascular system or cortical spreading depression.

### 5.4 Development of multi-omics integrated biomarker panels

Single ncRNA biomarkers often fail to meet the sensitivity and specificity requirements for clinical diagnosis; therefore, multi-omics integration strategies are key to constructing effective biomarker panels. Previous studies have combined miRNA expression data with genetic risk scores to build composite models for distinguishing migraine patients from healthy controls, achieving a positive predictive value exceeding 90% (13). However, this PPV was derived from a case–control study enriched with migraineurs versus healthy controls; PPV is prevalence-dependent, and this value may not reflect real-world clinical performance in the general population. Furthermore, Mendelian randomization analysis revealed causal associations between 169 CpG sites and migraine risk, involving genes such as CFDP1, ICA1L, and SERPING1, suggesting a potential interaction between DNA methylation and ncRNA regulation (40). At the functional level, the miR-653-3p/IGF1 axis contributes to migraine pathogenesis by regulating the AKT/TRPV1 pathway (24), while NEAT1 influences the expression of miR-196a-5p and Trpm3 via a ceRNA mechanism (3); however, this evidence is derived from a male-only mouse model, and the 3:1 female-to-male prevalence ratio of migraine in humans limits the direct generalizability of these findings; these pathway insights can guide the construction of multi-omics panels. Ideally, a biomarker panel should integrate miRNA, lncRNA, and circRNA expression profiles, combined with genetic variants (such as non-coding region SNPs like VAMP2*rs1150 and SNAP25*rs2327264 (28)), epigenetic modifications, and clinical phenotype data, and be optimized using machine learning algorithms. For example, machine learning models based on the oral microbiome have achieved an AUC of 0.83–0.88 for distinguishing migraine states (41); similar approaches can be extended to ncRNA multi-omics data to develop precision diagnostic tools suitable for predicting different migraine subtypes, chronicity risk, and drug response.

## 6 Potential targets for therapeutic interventions

### 6.1 Therapeutic prospects of RNA interference technology

In recent years, the therapeutic potential of RNA interference (RNAi) technology in neurological diseases has garnered increasing attention. In migraine research, experimental evidence has shown that RNA interference can effectively regulate the expression of key non-coding RNAs, thereby alleviating related symptoms. For example, in a male mouse model, the expression of the long non-coding RNA NEAT1 in the trigeminal ganglion was significantly upregulated and closely associated with photophobic behavior; following RNA interference using an shNEAT1 adeno-associated virus vector, NEAT1 expression was effectively downregulated, and photophobic-like behavior was markedly reduced. Mechanistic studies have shown that NEAT1 acts as a competitive endogenous RNA (ceRNA) to sequester miR-196a-5p, thereby upregulating Trpm3 expression and participating in the process of pain sensitization (3). This evidence is derived from a male-only mouse model; the 3:1 female-to-male prevalence ratio of migraine in humans limits the direct generalizability of these findings. Furthermore, differentially expressed miR-342-3p, miR-532-3p, and miR-758-5p in peripheral blood mononuclear cells have been shown to target genes related to ion channels, neurotransmitter and hormone homeostasis, and circadian rhythm regulation; these miRNAs may serve as ideal targets for RNA interference strategies (5). Furthermore, 5 and 31 differentially expressed miRNAs were identified in PBMCs from migraine patients during the attack phase and the interictal phase, respectively. Their target genes are enriched in immune inflammatory responses, neuroinflammation, and oxidative stress pathways, providing a molecular basis for the development of stage-specific RNAi therapies (2). These findings collectively support the feasibility and potential of RNA interference technology for precision intervention in migraine.

### 6.2 Precision regulatory strategies for epigenetic modification

Non-coding RNAs not only participate in gene expression as regulatory molecules but also influence migraine-related pathways by mediating epigenetic modifications. Studies have shown that the epigenetic status of the TRPA1 and CGRP genes is regulated by specific non-coding RNAs, and the RONS-TRPA1-CGRP signaling pathway plays a central role in the pathophysiology of migraine. While ncRNA-based approaches may offer sequence-specific targeting of this pathway, it is important to note that several CGRP-targeted therapies are already in clinical use, including monoclonal antibodies (erenumab, fremanezumab, galcanezumab) and small-molecule gepant-class receptor antagonists (rimegepant, ubrogepant, atogepant). These established drugs directly target the CGRP pathway at the protein or receptor level, demonstrating that ncRNAs are not the only therapeutic strategy capable of modulating this axis. Rather, ncRNA-based approaches represent a complementary strategy that may offer advantages in terms of upstream pathway regulation and multi-target effects (9). Furthermore, Mendelian randomization analysis identified 169 CpG sites with causal effects on migraine risk, involving 68 genes including CFDP1, ICA1L, and SERPING1, among others. Among these, MAPT and CALCA were identified as high-confidence potential therapeutic targets, suggesting a causal role for DNA methylation modifications in migraine pathogenesis and providing a theoretical basis for epigenetic editing (e.g., CRISPR/dCas9-fused methyltransferases or demethylases) (40). Concurrently, histone modifications also participate in regulating key inflammatory genes. For example, in LPS-stimulated microglial cells, the lncRNA Nostrill exacerbates neuroinflammation by promoting the recruitment of NF-κB p65 and RNA polymerase II to the iNOS promoter region and enhancing H3K4me3 histone modification; silencing Nostrill significantly reduces iNOS expression and neurotoxicity (42). These findings suggest that targeting non-coding RNA-mediated epigenetic mechanisms holds promise for the precise reprogramming of migraine-associated gene networks.

However, several critical limitations must be acknowledged regarding ncRNA-based therapeutic strategies. First, off-target effects represent a major concern: both siRNAs and antisense oligonucleotides can produce unintended gene silencing through partial sequence complementarity, potentially affecting non-target transcripts. For miRNA-based therapeutics, the pleiotropic nature of miRNAs—which typically regulate hundreds of target genes simultaneously—poses challenges for specificity. Second, safety concerns include potential immune stimulation (e.g., Toll-like receptor activation by exogenous RNA), hepatotoxicity, and the risk of disrupting endogenous ncRNA regulatory networks. Third, no ncRNA-based therapeutic for migraine has entered clinical trials to date; all current evidence is derived from preclinical models, and the path to clinical translation remains uncertain. These limitations underscore the need for rigorous safety evaluation and long-term follow-up in future translational studies.

### 6.3 RNA regulatory mechanisms of existing drugs

Existing migraine treatments may exert their therapeutic effects through indirect regulation of non-coding RNAs; this “new mechanism for old drugs” perspective offers new insights for drug repurposing. Clinical observations have shown that following treatment with CGRP receptor antagonists of the gepant class, the abnormally elevated levels of certain miRNAs (such as miR-145-5p and miR-26a-5p) in patients’ serum tend to decline, suggesting that these miRNAs may not only serve as disease biomarkers but also participate in drug action pathways, and their dynamic changes may be used to assess treatment response (11). Furthermore, many prophylactic drugs possess antioxidant properties, and oxidative stress is believed to play a key role in the chronicization of migraine (43); given that the miRNA expression profile is significantly enriched in oxidative stress pathways during attack episodes (2), it is speculated that existing antioxidant drugs may exert their effects in part by regulating related miRNA networks. Notably, non-coding variants such as VAMP2*rs1150, SNAP25*rs2327264, and STX1A_rs6951030 may contribute to migraine susceptibility by influencing the expression of SNARE complex genes; the mechanism may involve altering transcription factor binding or miRNA targeting efficiency (28). This further suggests that targeting these non-coding regions for regulation may optimize the personalized application of existing drugs. Therefore, a systematic analysis of the effects of existing drugs on non-coding RNA networks can help elucidate their underlying mechanisms of action and guide combination therapy strategies.

### 6.4 Delivery systems and blood–brain barrier penetration technologies

Although RNA interference and epigenetic editing strategies have shown potential in migraine treatment, their clinical translation faces challenges posed by blood–brain barrier (BBB) limitations and low targeted delivery efficiency. Currently, studies have utilized viral vectors to achieve effective regulation of non-coding RNAs within the central nervous system. For example, adeno-associated virus (AAV) vectors have been successfully used to deliver shNEAT1 to the trigeminal ganglion in mice, achieving local NEAT1 knockdown and improving behavioral phenotypes (3). This evidence is derived from a male-only mouse model; the 3:1 female-to-male prevalence ratio of migraine in humans limits the direct generalizability of these findings. Additionally, exosomes, as natural nanocarriers, have garnered significant attention in the treatment of neurological diseases. In a TBI mouse model, exosomes derived from astrocytes can transport the long non-coding RNA 4933431K23Rik into microglia, where they suppress the NF-κB pathway via the miR-10a-5p/E2F7/TFAP2C axis, thereby alleviating neuroinflammation (16). This finding was derived from a traumatic brain injury (TBI) mouse model, not a migraine model; its relevance to migraine is speculative and requires direct validation. This mechanism suggests that engineered exosomes hold promise for delivering therapeutic non-coding RNAs or siRNAs to migraine-related brain regions. Meanwhile, CRISPR/Cas13d vectors based on the PiggyBac transposon system have achieved RNA knockdown efficiencies as high as 98% in human pluripotent stem cells (44). Although not yet applied in migraine models, this provides a technical foundation for developing highly efficient RNA editing tools. In the future, it will be necessary to further optimize the BBB penetration capacity of non-viral delivery systems (such as lipid nanoparticles and polymeric carriers) and combine them with targeting ligands (such as transferrin receptor antibodies) to enhance the specificity of delivery to the trigeminovascular system or cortical regions, thereby advancing non-coding RNA-targeted therapies from the laboratory to clinical practice.

## 7 Current challenges and future directions

### 7.1 Technical bottlenecks in tissue-specific and spatiotemporal dynamic analysis

A core challenge in current non-coding RNA research on migraine lies in the highly tissue-specific and spatiotemporal dynamic nature of its expression, while existing technical methods still struggle to achieve high-resolution, dynamic monitoring in key target tissues such as the living human brain or trigeminal ganglia. For example, although previous studies have identified migraine-associated genes such as ATG7 through single-cell RNA sequencing of human trigeminal ganglia combined with Mendelian randomization analysis—where its upregulation promotes the activation of autophagy—and these studies suggest that non-coding RNAs may be involved in the pathophysiological mechanisms of migraine (31), the difficulty in obtaining such samples and strict ethical constraints severely limit the in-depth analysis of dynamic changes in ncRNAs within the central nervous system. Furthermore, in animal models, the long non-coding RNA NEAT1 has been observed to be upregulated in the trigeminal ganglia of mice, where it regulates the expression of miR-196a-5p and Trpm3 via a ceRNA mechanism, thereby influencing photophobic behavior (3); however, this evidence is derived from a male-only mouse model, and the 3:1 female-to-male prevalence ratio of migraine in humans limits the direct generalizability of these findings. Meanwhile, although differential miRNA expression detected in peripheral blood mononuclear cells (PBMCs)—such as hsa-miR-3202 and hsa-miR-7855-5p—is associated with the active and interictal phases (2), it remains uncertain whether these findings truly reflect molecular events within the central nervous system. Therefore, establishing a technical system capable of crossing the blood–brain barrier to accurately capture dynamic changes in ncRNA in target tissues remains a critical bottleneck requiring urgent breakthroughs.

### 7.2 Standardization and validation systems for clinical translation research

Although multiple studies have identified potential migraine-associated non-coding RNA biomarkers—such as miR-155, which is significantly elevated in patients with chronic migraine with medication overuse and positively correlated with pro-inflammatory factors IL-1β and TNF-*α* (1), or miR-145-5p and miR-26a-5p, which show elevated levels in patient serum (6)—these findings have not yet led to the establishment of unified testing standards or clinical validation pathways. Current studies predominantly employ small-sample cross-sectional designs, and sample processing procedures (such as blood collection timing, RNA extraction methods, and sequencing platforms) lack consistency, resulting in limited reproducibility of results. For example, although a prospective cohort study confirmed the abnormal expression of miR-183, miR-25, and miR-320 in migraine patients in both the discovery cohort (*n* = 120) and the validation cohort (*n* = 197), and constructed a composite model with a positive predictive value exceeding 90% (13), this PPV was derived from a case–control study enriched with migraineurs versus healthy controls; PPV is prevalence-dependent, and this value may not reflect real-world clinical performance in the general population. Furthermore, this model has not yet been externally validated in an independent, multicenter population. Furthermore, the phenomenon where treatment response-related biomarkers, such as hsa-miR-143-3p, decrease as efficacy increases following erenumab treatment (12), also requires standardized evaluation in larger-scale, prospective intervention trials. Therefore, there is an urgent need to establish a standardized system covering the entire process—from sample collection and stable RNA preservation to detection platform calibration and data analysis—and to promote a multi-stage validation framework (discovery–validation–clinical application) based on international consensus.

### 7.3 The need to establish multicenter, large-sample cohorts

Existing studies on the association between non-coding RNA and migraine generally suffer from small sample sizes and high population heterogeneity. For example, some studies included only 13 patients and controls (6), or were limited to specific gender groups (such as premenopausal women) (5), making it difficult to represent the molecular characteristics of the overall migraine population. Similarly, the circRNA profiling study by Lin et al. (2020) included only 4 migraine patients and 3 controls (45), and the lncRNA study by Taheri et al. (2024) examined a relatively small cohort (46), underscoring the need for larger-scale validation. Although previous studies have attempted to distinguish episodic migraine (EM) from chronic migraine (CM), and found that miR-155 levels are significantly higher in CM-MO patients than in EM patients (1), the robustness of the differential expression profiles between subtypes still requires support from large-scale data. Furthermore, the influence of genetic background on ncRNA expression cannot be overlooked; for example, the rs12355831: A > G variant in European populations has been shown to affect migraine risk by regulating ACSL5 splicing (41), but it remains unclear whether this mechanism is conserved in other ethnic groups. Therefore, there is an urgent need to establish large-scale, multicenter cohorts covering different regions, ethnicities, genders, ages, and clinical phenotypes (e.g., with/without aura, chronic status, drug responsiveness). Such cohorts should integrate clinical phenotypes, imaging, genetic information, and multi-omics data to support hierarchical analysis and causal inference of ncRNA biomarkers, laying the foundation for precise subtyping and personalized interventions.

### 7.4 Application prospects of single-cell technologies and spatial transcriptomics

With the advancement of single-cell RNA sequencing (scRNA-seq) and spatial transcriptomics, it has become possible to elucidate the cell-type-specific expression and spatial distribution of non-coding RNAs in migraine. Previous studies have utilized single-cell sequencing data from human trigeminal ganglia, combined with cortical cell-type eQTL information, to reveal co-expression patterns of genes such as ATG7 in both the central and peripheral nervous systems (31), providing clues for subsequent functional characterization of ncRNAs. In the future, scRNA-seq could further identify differentially expressed lncRNAs, circRNAs, or miRNAs in specific neuronal and glial cell subpopulations within the trigeminal ganglion, brainstem pain modulation nuclei, or the cortex. For example, it may be possible to determine whether NEAT1 is specifically enriched in sensory neurons and regulates TRPM3 channels (3); however, this evidence is derived from a male-only mouse model, and the 3:1 female-to-male prevalence ratio of migraine in humans limits the direct generalizability of these findings. Spatial transcriptomics holds promise for revealing the dynamic changes in ncRNA expression within the cortical laminar structure during cortical spreading depression (CSD), thereby elucidating its role in the onset of aura. Furthermore, by combining exosome isolation with single-cell multi-omics technologies, it is possible to trace whether ncRNAs in the peripheral circulation originate from specific central cell types, thereby enhancing their reliability as “liquid biopsy” biomarkers. These cutting-edge technologies will drive a paradigm shift from “tissue-averaged signals” to “cell-resolution regulatory networks,” providing unprecedented resolution for elucidating the regulatory mechanisms of ncRNAs in migraine (3, 31).

## 8 Summary and discussion

In recent years, significant progress has been made in studying the role of non-coding RNAs (ncRNAs) in the pathogenesis of migraine, driving a systematic reconceptualization of the disease’s molecular basis. Multiple studies have demonstrated that microRNAs, long non-coding RNAs (lncRNAs), and circular RNAs (circRNAs) are deeply involved in the pathophysiological processes of migraine by regulating key signaling pathways, neurovascular function, and inflammatory responses. For example, miR-145-5p, miR-26a-5p, and miR-19a-3p exhibit attack-state-dependent expression changes in patient serum, suggesting a close association with the dynamic course of migraine (6). Analysis of the miRNA expression profile in peripheral blood mononuclear cells (PBMCs) further revealed that 31 miRNAs were differentially expressed during the interictal period, while 5 showed significant changes during the attack phase. These miRNAs are enriched in immune inflammation, neuroinflammation, and oxidative stress pathways, and their target genes are highly consistent with mRNA transcriptomic data, establishing the first migraine-specific ncRNA regulatory network (2). Furthermore, the lncRNA NEAT1 is upregulated in the trigeminal ganglion and regulates miR-196a-5p and Trpm3 via a ceRNA mechanism; its knockdown alleviates photophobic behavior, highlighting the critical role of ncRNAs in sensory sensitization (3). However, this evidence is derived from a male-only mouse model, and the 3:1 female-to-male prevalence ratio of migraine in humans limits the direct generalizability of these findings. Recent studies have also provided direct evidence for circRNA involvement in migraine, including differential circRNA expression profiles in patient PBMCs (7) and functional characterization of hsa_circ_0006168 in promoting central sensitization via the miR-99b-5p/KDM6B axis (8). Additionally, the lncRNAs PVT1 and MEG3 have been shown to be differentially expressed between migraine with and without aura (23), expanding the known lncRNA landscape beyond NEAT1. At the molecular level, ncRNA also regulates calcitonin gene-related peptide (CGRP) production by influencing alternative splicing of the CALCA gene, thereby activating the TRPA1 and MAPK1/2 pathways, forming a core regulatory link in the “RONS-TRPA1-CGRP” axis (9). Concurrently, METTL3-mediated m6A modification enhances miR-34a-5p expression, inhibits the Wnt1/*β*-catenin pathway, and promotes activation of the trigeminovascular system, further expanding the regulatory dimensions of the epigenome in migraine (10). These findings collectively indicate that ncRNAs not only integrate genetic, environmental, and neurovascular signals as upstream regulatory factors but also drive the onset and progression of migraine through multi-level interactive networks.

Importantly, the recent incorporation of human clinical miRNA evidence has enabled cross-validation of findings originally derived from animal and high-throughput profiling studies. Early profiling efforts identified distinct miRNA signatures in plasma exosomes (15) and serum (16), and a key convergence subsequently emerged around miR-382-5p and miR-34a-5p, which were independently identified as upregulated during migraine attacks in serum (16), elevated in PBMCs and monocytes of chronic migraine patients (18, 20), and reduced following anti-CGRP therapy (21). This cross-matrix consistency—spanning serum, PBMCs, and purified monocytes—suggests that these miRNAs may reflect a fundamental disease process rather than a compartment-specific artifact. At the mechanistic level, human studies have validated the CGRP–miRNA regulatory axis previously characterized in preclinical models: miR-30a was shown to directly target CALCA mRNA (encoding *α*-CGRP) in patient peripheral blood (17), providing the first post-transcriptional regulatory link between miRNAs and the CGRP pathway. Additionally, the elevation of endothelial-specific miRNAs (miR-155, miR-126, let-7g) in migraine patients’ plasma (19) extends miRNA involvement beyond neuroinflammation to vascular mechanisms, complementing the predominantly neuroinflammatory focus of existing preclinical models. However, it should be acknowledged that the majority of these human studies employed small cohorts (15–47 patients per group) and cross-sectional designs, with several key findings awaiting independent replication by other research groups.

Based on current evidence, non-coding RNAs demonstrate significant potential as biomarkers and therapeutic targets for migraine; however, their clinical translation requires a well-defined pathway. First, efforts should focus on establishing standardized detection systems, particularly by screening for highly specific ncRNA biomarkers in readily accessible samples such as peripheral blood, cerebrospinal fluid, and exosomes. Studies have confirmed that a triplet combination comprising miR-342-3p, miR-532-3p, and miR-758-5p can effectively distinguish migraine patients from healthy controls; their target genes are involved in ion channel and neurotransmitter homeostasis, providing a foundation for serving as reliable epigenetic markers (5). Additionally, miR-155 is significantly elevated in patients with chronic migraine with medication overuse and is positively correlated with pro-inflammatory factors such as IL-1β and TNF-*α* (detailed values presented in Section 2.1) (1). A notable methodological advance is the proposal of a combined CGRP + miRNA biomarker panel, which integrates neuropeptide and epigenetic markers into a single diagnostic framework (18). This approach is conceptually distinct from single-analyte biomarker strategies and may offer improved discriminative power for distinguishing migraine phenotypes, as evidenced by the correlation between CGRP and miR-382-5p/miR-34a-5p levels (Spearman’s rho = 0.491 and 0.303, respectively) and their concordant reduction following detoxification. Furthermore, the demonstration that miR-382-5p and miR-34a expression in purified monocytes showed more pronounced disease-associated changes than in bulk PBMCs (20) highlights the importance of cell-type-specific resolution for biomarker development—a consideration that future assay standardization efforts must address. Furthermore, there is a need to develop multi-omics integration strategies that combine ncRNA expression profiles with genetic risk scores; for example, a composite model integrating miRNA and genetic risk scores has achieved a positive predictive value of >90%, significantly enhancing diagnostic performance (13). However, this PPV was derived from a case–control study enriched with migraineurs versus healthy controls; PPV is prevalence-dependent, and this value may not reflect real-world clinical performance in the general population. Furthermore, dynamic monitoring of ncRNA changes is crucial for assessing treatment response: following erenumab treatment, the expression levels of hsa-miR-143-3p, hsa-miR-34a-5p, and hsa-miR-382-5p decreased as clinical efficacy improved, indicating that they can serve as dynamic markers for predicting drug responsiveness (12). This finding has been independently corroborated by De Icco et al. (21) in an open-label study of 40 chronic migraine patients, where erenumab significantly reduced miR-382-5p (*p* = 0.015) and miR-34a-5p (*p* = 0.001) levels in PBMCs (21). However, the absence of a placebo arm limits causal attribution, and the finding that miRNA changes did not differ significantly between clinical responders and non-responders raises the possibility that miRNA reduction reflects a general pharmacological effect of CGRP pathway blockade rather than a specific marker of treatment efficacy. This nuance has implications for biomarker interpretation: miRNAs may serve as pharmacodynamic markers of target engagement, but their utility as predictors of clinical response requires further validation in controlled trials. Moreover, the reduced upregulation of miRNAs following treatment with gepants further supports the potential application of ncRNAs in personalized therapy (11). Therefore, future efforts should focus on advancing prospective cohort studies to validate the stability and universality of candidate ncRNAs across different subtypes, disease stages, and treatment contexts, thereby providing evidence-based support for clinical decision-making.

In-depth exploration of non-coding RNAs in migraine research urgently requires interdisciplinary collaborative innovation, integrating the technical strengths of multiple fields such as genomics, epigenetics, neuroscience, and bioinformatics. On the one hand, the integration of high-throughput sequencing with functional validation should be strengthened; for example, the integration of trigeminal ganglion single-cell RNA sequencing with cortical eQTL data, combined with Mendelian randomization analysis, has successfully identified key genes such as ATG7 that exert both central and peripheral effects (47); similar strategies can be extended to causal inference in ncRNA-target gene networks. On the other hand, the application of spatial transcriptomics and single-cell technologies will help elucidate the spatiotemporal dynamics of ncRNA expression in specific neuronal subpopulations or glial cells, overcoming the current interpretation bottlenecks caused by tissue heterogeneity. Furthermore, drawing on experience from the field of oncology, methods for the enrichment and detection of exosomal ncRNAs (such as the differentiation-antagonistic non-coding RNA used for hepatocellular carcinoma prognosis (32)) can be adapted to migraine research, thereby enhancing the sensitivity and specificity of biomarkers. At the therapeutic level, RNA interference and epigenetic editing tools (such as targeting LINC00665 to restore gefitinib sensitivity (38)) provide technical paradigms for the precise regulation of ncRNAs; however, these must be combined with blood–brain barrier-penetrating delivery systems to achieve central nervous system targeting. Critically, the absence of clinical trials, potential off-target effects, and safety concerns associated with ncRNA-based therapeutics must be addressed before translational applications can be contemplated. Finally, establishing multicenter, large-scale cohorts with detailed phenotypic stratification (e.g., distinguishing between aura/non-aura and episodic/chronic subtypes) and standardizing sample collection, processing, and data analysis protocols are the cornerstones for advancing ncRNA from mechanistic discovery to clinical application. In particular, the recently reviewed human miRNA literature highlights several methodological gaps that future studies must address: (1) sample sizes remain small (15–47 patients per group), limiting statistical power and generalizability; (2) the absence of standardized preanalytical protocols across studies—encompassing diverse biological matrices (serum, plasma, exosomes, PBMCs, monocytes), collection methods, and normalization strategies—hampers cross-study comparison and meta-analysis; (3) most studies employ cross-sectional rather than longitudinal designs, precluding assessment of intra-individual miRNA variability over time; and (4) three of the seven reviewed studies (18, 20, 21) originate from the same research group, raising the possibility of systematic bias. Addressing these gaps through large-scale, multicenter, longitudinal cohorts with harmonized protocols is essential for advancing candidate miRNA biomarkers toward clinical implementation. Only through multidimensional collaboration across basic research, clinical practice, and engineering can the transformative potential of non-coding RNA in migraine precision medicine be fully realized.

## Glossary

NcRNA: non-coding RNA
miRNA: microRNA
lncRNA: long non-coding RNA
circRNA: circular RNA
CM-MO: chronic migraine with medication overuse
PBMC: peripheral blood mononuclear cell
IL: interleukin
TNF: tumor necrosis factor
NEAT1: nuclear paraspeckle assembly transcript 1
ceRNA: competing endogenous RNA
Trpm3: transient receptor potential cation channel subfamily M member 3
CSD: cortical spreading depression
CGRP: calcitonin gene-related peptide
TRP: transient receptor potential
RONS: reactive oxygen and nitrogen species
TRPA1: transient receptor potential ankyrin 1
snoRNA: small nucleolar RNA
tRF: tRNA-derived fragment
m^6^A: N6-methyladenosine
EM: episodic migraine
CM: chronic migraine
RCVS: reversible cerebral vasoconstriction syndrome
METTL3: methyltransferase-like 3
Wnt: wingless-related integration site
MWA: migraine with aura
MO: migraine without aura
MA: migraine with aura
NO: nitric oxide
AKT: protein kinase B
TRPV1: transient receptor potential vanilloid 1
ATG7: autophagy-related gene 7
LC3: microtubule-associated protein 1 light chain 3
NLRP3: NLR family pyrin domain containing 3
KP: kynurenine pathway
AhR: aryl hydrocarbon receptor
GPR35: G protein-coupled receptor 35
PACAP: pituitary adenylate cyclase-activating polypeptide
TNC: trigeminal nucleus caudalis
GABA: gamma-aminobutyric acid
GAD65: glutamate decarboxylase 65
CSF: cerebrospinal fluid
eQTL: expression quantitative trait locus
SNP: single nucleotide polymorphism
AUC: area under the curve
PPV: positive predictive value
RNAi: RNA interference
shRNA: short hairpin RNA
AAV: adeno-associated virus
CRISPR: clustered regularly interspaced short palindromic repeats
dCas9: catalytically dead Cas9
BBB: blood–brain barrier
scRNA-seq: single-cell RNA sequencing
qPCR: quantitative polymerase chain reaction
MAPK: mitogen-activated protein kinase
EZH2: enhancer of zeste homolog 2
PI3K: phosphoinositide 3-kinase
PVT1: Pvt1 oncogene
MEG3: maternally expressed gene 3
TBI: traumatic brain injury
KDM6B: lysine demethylase 6B
